# Medical Items

**Published:** 1898-01

**Authors:** 


### *MEDICAL ITEMS.*

---

DR. JOSEPH L. ESTES, of Eastman, Georgia, died December 17th.

---

DR. MARCUS L. CARSON, of Griffin, is spending the winter in London.

---

DR. DON B. BOSWORTH, of Atlanta, died December 28th, in the twenty-eighth year of his age.

---

DR. V. H. TALLIAFERRO, of Atlanta, and Miss Leverett, of Covington, were married December 22d.

---

THANKS to a kind Providence, we again have the pleasure of wishing all our readers and patrons a happy and prosperous New Year.

---

DR. EDWARD B. JACKSON, of Houston, Texas, desires a young physician as partner; must be energetic and sober. Write or call for particulars.

---

THE *Hot Springs Medical Journal* says that a negro doctor in an Alabama town has at the top of his professional card: "No pay, no cure."

---

WHAT is fame? A daily paper reporting the recent meeting of the British Medical Society mentions the celebrities in attendance, among whom was Lord Lister, of Listerine fame.—*Ex.*

---

THE two schools, the Southern and the Atlanta Medical College, both closed during the Christmas holidays. The clinics, however, continue without interruption. Both schools report large classes.

---

DR. W. A. STARNES, of Jackson, Georgia, was married to Miss Hattie Marbut, of Atlanta, December 8th. Dr. Starnes is one of the coming physicians in his section, and we wish him and his wife all possible success.

---

ON Wednesday night, December 29th, the annual dinner of the Medical Board of the Grady Hospital occurred at the Aragon Hotel. Dr. W. S. Elkin was elected President, and Dr. J. G. Earnest, Secretary, for the year 1898.

---

How is this for progressive surgery? Professor of surgery in an Eclectic College (not a thousand miles from Atlanta) to his class: "Antisepsis is all rot. I had as soon wrap up a wound in an old dirty shirt as in antiseptics. The result would be the same."

---

GOVERNOR ATKINSON vetoed the bill which was passed by the legislature of Georgia prohibiting football playing in that State. The Governor gave as his reason that the faculties of the colleges should regulate this question. The Governor is wrong.—*Alabama Medical and Surgical Age.*

---

BEGINNING this month a foreign edition of *The Laryngoscope* will be published in Bristol, England, by John Wright & Co. This is a well-deserved compliment to one of the newest, and one of the best of American medical periodicals, devoted to diseases of the nose, throat, and ear.

---

THE Grady Hospital has established a Training School for Nurses, regularly incorporated, with the power of issuing diplomas. Lectures are given to the nurses by the attending hospital staff of physicians. Miss Patterson, a graduate nurse from the Johns Hopkins Hospital, is in charge of the Training School.

---

WE call attention to the "Medical Reminiscences" of Bill Arp in this issue. This is the beginning of a series which we hope to publish at intervals during the year. Bill Arp's learning and popularity and his matchless acquaintance with men and matters in Georgia history will make this an exceedingly interesting feature of THE JOURNAL.

---

DURING the recent epidemic of yellow fever in New Orleans, twelve physicians were stricken with the disease, and two of them died. The same number of trained nurses were also attacked, but all recovered. The two physicians who succumbed to the disease were young men who had not lived long in New Orleans and were unacclimated.—*Med. News.*

---

THE fall term of Tulane University, at New Orleans, which was delayed on account of the prevalence of yellow fever in that city, was opened November 29th. The faculty has announced that the session will continue until June 20, 1898, with no vacations and only occasional intermissions on holidays, in order to get in a full year's work before the next commencement.

---

DR. I. C. ROSSE, of Washington, D. C., does not believe in hydrophobia, and he has offered a reward of $100 for a single well authenticated case of that disease in either dog or man. The *Texas Medical Practitioner* says that if the doctor will come out to Texas and "bite himsuf mit a Texas skunk," as the Dutchman says, he will see an authentic case to his entire satisfaction within two weeks.

---

OFFICERS of the Atlanta Society of Medicine for 1898: President, Dr. W. L. Champion; Vice-president, Dr. W. S. Goldsmith;

Secretary, Dr. L. A. Felder; Treasurer, Dr. L. B. Grandy; Librarian, Dr. Michael Hoke. Hereafter the Society will meet on the first and third Friday evenings of each month in the Knights of Pythias Hall, over Brown & Allen's drug store, 23½ Whitehall street.

---

THE ATLANTA MEDICAL AND SURGICAL JOURNAL, in commenting upon the prize offered by the Medical Society of Virginia for a history of medicine and surgery in Virginia, very pertinently asks the question: "Why can we not get a history of medicine and surgery in Georgia in a similar manner?" Why not, indeed; never was more reasonable question asked. Our medical history is a glorious one; by all means let us have it. The editor of THE ATLANTA JOURNAL has already given us a résumé of a history of medicine in this State and a most excellent work it is. We will join hands with THE ATLANTA JOURNAL in urging this matter at the next State meeting, and meanwhile let it be discussed in all the local societies.—*Georgia Journal of Medicine and Surgery.*

---

ACCORDING to a report in the daily papers, Col. E. E. Cavaleri, a veteran of the Union army, seventy-four years of age, has sued the city [Atlanta] for damages. When the city physicians were recently making their vaccination rounds Col. Cavaleri's *fiancée* objected to vaccination, saying she was going to be married. The doctor told her it was better to be vaccinated than marry. When Cavaleri came next to see her she told him she had decided to follow the doctor's advice, and refused to marry him. Hence the suit for damages.—*Boston Medical and Surgical Journal, December 16, 1897.*

Some amateur fiction-artist has evidently imposed upon our esteemed Boston contemporary. We have heard nothing of the unfortunate case above related. Neither has the Board of Health. The author of the above fake-despatch must have been entertaining his Christmas jag somewhat in advance of the season.

"APENTA" is the title of a little brochure just sent us by The Apollinaris Company, and containing reports and opinions from distinguished physicians and chemists upon the properties and virtues of this (natural) aperient water. Among these authorities we note Professors Liebrich and Gerhardt of Berlin, Professor Pouchet of Paris, Professor Tichborne of Dublin, Professor Althaus of London, and others. The "Apenta" Springs are under the control of Professor Fodor, Director of the Hygienic Institute, Royal University, Buda-Pest, and the water is said to be "bottled under eminent scientific supervision, so as to insure its constancy of composition and strength, and its freedom from all impurities." Those interested in aperient waters can procure the booklet from I. Haldenstein, Forty-second Street and Fifth Avenue, New York.

---

THE small epidemic of smallpox which has been prevailing in Atlanta for the last three months is now about at an end. The first case made its appearance on the 20th of August, and in the next few days several other cases were found. These appeared to be all, but early in October a second series of cases were discovered, and from these a general infection of the city was threatened. A corps of doctors was employed to vaccinate the city, particularly among the negroes. The physicians went over the city twice and did thorough and efficient work, and they doubtless averted a serious epidemic. There have been altogether 232 cases; 94 have been discharged cured, and two have died. There were 203 negroes, and 29 whites. Nine patients now in the smallpox hospital have confluent smallpox and are very ill. It is thought that one will die.

The smallpox hospital consists of six well built houses, in which patients are thoroughly protected from the weather and made as comfortable as possible in the circumstances. Dr. J. L. Stockdell has been resident physician at the hospital for four months. Patients have been treated with all due care and attention. The very low mortality speaks for itself.

---

AT the call of Governor Johnston, of Alabama, a conference was held in Mobile, December 18th, looking to the holding of a quarantine convention of the South Atlantic and Gulf States for the better protection of the people from the invasion of the disease and from the spread of the same.

The meeting was suggested by State Health Officer Sanders, and was attended by representatives of several States.

Representation was fixed at five members, appointed by the governor of each State, one delegate from each municipality, and one from each commercial organization, railroad system, and river transportation company, all chiefs of quarantine service in the States to be invited. The program covers the whole subject of quarantine in relation to State and national government; the problem of disinfection and sanitation, regulations concerning transportion of freight and passengers; national versus State control of quarantine; the proposed national bureau of public health, etc.

On motion of Dr. Olliphant, of the Louisiana Board of Health, the chairman, Governor Johnston, appointed an Executive Committee of nine to prepare for and call the convention at such time and place as will be most convenient. The names of the committeemen are:

President of the Louisiana Board of Health, State Health Officer Sanders, of Alabama; Porter, of Florida; Dr. Harralson, of Mississippi; Mayor Bush, of Mobile; Mayor Flower, of New Orleans; the Mayor of Pensacola, ex-Governor Stone, of Mississippi, and the President of the Mobile Board of Health, Dr. Ketchum.

A resolution was adopted appealing to Congress to withhold action on the public health and the quarantine matters until the subject can receive the attention its importance demands.

